# Probiotic Potential of Lactobacilli Isolated from Saliva of Periodontally Healthy Individuals

**DOI:** 10.3290/j.ohpd.a44693

**Published:** 2020-07-04

**Authors:** Masaaki Hirasawa, Tomoko Kurita-Ochiai

**Affiliations:** a Postgraduate, Department of Microbiology and Immunology, Nihon University School of Dentistry at Matsudo, Matsudo, Chiba, Japan. Wrote the manuscript, performed the experiments, contributed substantially to discussion.; b Professor, Department of Microbiology and Immunology, Nihon University School of Dentistry at Matsudo, Matsudo, Chiba, Japan. Set up the experimental design, wrote the manuscript, consulted on and performed statistical evaluation, contributed substantially to discussion.

**Keywords:** antibacterial substance, lactobacilli, oral cavity

## Abstract

**Purpose:**

To search for useful probiotics, we characterised antimicrobial lactic acid bacteria isolated from the oral cavities of 20 healthy volunteers from Nihon Univerisity School of Dentistry.

**Materials and Methods:**

Oral lactobacilli were isolated from the saliva of 20 periodontally healthy volunteers using the Dentocult LB dip-slide method. Primary antimicrobial screening of *Lactobacillus* isolates was performed using the paper disk method. Each suspected antimicrobial isolate was tested against *Streptococcus mutans, S. sobrinus*, and *Porphyromonas gingivalis*. Since *P. gingivalis* is considered as a keystone bacterium, we further analysed lactic acid bacteria that produced extracellular soluble antimicrobial agents that created an inhibitory zone of more than 12 mm against *P. gingivalis*. After two rounds of antimicrobial susceptibility testing, six isolates showing strong antibacterial effects against *P. gingivalis* were selected and identified as facultatively anaerobic, Gram-positive, non-spore-forming, non-capsule-forming, and catalase-negative bacilli. Selected isolates were identified to the species level through 16S rDNA sequence analyses.

**Results:**

The antimicrobial substance produced by the isolated bacilli was inactivated slightly after catalase treatment, had significantly lower activity at pH levels above 9.0, and was also reduced by heat treatment above 100°C and autoclaving. The activity of the antimicrobial substance showed some resistance to lower levels of heat, as well as pepsin and proteinase K treatments. 16S rRNA sequencing identified these isolates as *Lactobacillus casei, Lactobacillus fermentum,* and *Lactobacillus gasseri*.

**Conclusion:**

These bacteria are antagonistic against potential periodontal pathogens and are good candidates for clinical application as probiotics.

Dental caries and periodontal disease are infectious diseases associated with dysbiosis of the microorganisms residing in dental plaque biofilms.^22^ *Porphyromonas gingivalis* is thought to be a keystone bacterium in developing periodontal disease, whereas *Streptococcus mutans* and *S. sobrinus* are clearly associated with caries. Recent epidemiological research has suggested that oral bacteria are also associated with many systemic diseases including pneumonia, cardiovascular disease, diabetes, and rheumatoid arthritis.^19,29^

Lactobacillus species have been found in the microbiota of the mouth, intestine, and vagina of humans.^1^ As probiotics, they play an important role in the maintenance of health by stimulating natural immunity and contributing to the balance of microflora through interactions with other microbes.^23,27,34^ Lactobacilli produce organic acids such as lactic acid and acetic acid from carbohydrate fermentation, leading to low ambient pH that can interfere with the growth of surrounding microorganisms. In addition, some species of lactobacilli produce hydrogen peroxide or bacteriocins, which are known antimicrobial substances.^8,15,24,36^ Due to their antimicrobial and interference activities, lactobacilli have inhibitory effects against a variety of microorganisms. Several studies have investigated the roles of lactobacilli in protection against oral,^16,17^ intestinal, and vaginal infections.^11,25,26^ Based on their antimicrobial properties, these bacteria may be beneficial as bioprotective agents for control of infections in the mouth, intestine, and vagina. Therefore, in order to investigate to what extent *Lactobacillus* species isolated from the oral cavities of volunteers with good periodontal health possess antibacterial activity against cariogenic and periodontopathic bacteria, the antimicrobial activities against *S. mutans, S. sobrinus* and *P. gingivalis* underwent primary screening. Furthermore, among the ten strains having antibacterial activities against *P. gingivalis* as a keystone pathogen for periodontal disease, six strains with particularly high antibacterial activity were identified. We also examined the effects of enzymes, pH, and heat on the antibacterial factors produced by these *Lactobacillus* species using *P. gingivalis* as an index.

## Materials and Methods

### Isolation of the Suspected Strains

Oral lactobacilli were isolated from the saliva of 20 volunteers without periodontal health problems from our dental school using the Dentocult LB dip-slide (Orion Diagnostica; Espoo, Finland) method.^5^ These volunteers showed no radiographic or clinical evidence of attachment loss. Furthermore, we measured the gingival index^21^ of each subject to confirm the absence of gingivitis. No notable increase in gingival inflammation was observed in any subject. Informed consent was obtained from all subjects in accordance with the procedures of the Ethics Review Committee on Human Research of the Nihon University School of Dentistry at Matsudo (ECO 2–015). Paraffin-stimulated whole saliva was collected, and a 1-ml aliquot was transferred to a selective dip-slide. The slides were incubated at 37°C using a model 1024 anaerobic system (Forma Scientific; Marietta, OH, USA) with 10% H_2_, 80% N_2_, and 10% CO_2_ for 2–3 days. Provisional identification of *Lactobacillus* species and strains was based on colony and cell morphologies. Colonies with different morphologies were chosen from the Dentocult LB dip-slide and transferred to Rogosa SL agar (RSA, Difco; Franklin Lakes, NJ, USA) selective medium for lactobacilli to isolate *Lactobacillus* strains, and then incubated under anaerobic conditions for 24–48 h. Thereafter, the lactobacilli were repeatedly streaked onto a series of RSA plates to purify the culture. At each step of the purification process, both the colony and cell morphologies of the isolate were determined. All colonies were checked with Gram staining and catalase testing prior to further identification. All catalase-negative isolates that were suspected to be lactobacilli were selected for further study.

### Screening of Antimicrobial Isolates

Primary antimicrobial screening of the *Lactobacillus* isolates was performed using the paper disk method.^13^ The culture supernatant from each suspected antimicrobial isolate was tested against *Streptococcus mutans* PS-14, *S.* *sobrinus* 6715, *Porphyromonas gingivalis* FDC 381, and *P.* *gingivalis* ATCC 33277. These bacteria were maintained at the Nihon University School of Dentistry at Matsudo. *P. gingivalis* 381 and 33277 were pre-cultured in brain heart infusion (BHI) broth (Difco) supplemented with 5 µg/ml hemin (Wako Pure Chemical Industries; Osaka, Japan) and 0.4 µg/ml menadione (Wako Pure Chemical Industries), while the other pathogenic bacteria were pre-cultured in BHI broth at 37°C for 24 h under anaerobic conditions. These bacterial strains were adjusted to approximately 1 x 10^8^ colony forming units (CFU)/ml, then dropped onto blood agar plate in volumes of 100 µl and spread using a sterile glass spreader. The *Lactobacillus* isolates were grown in Rogosa SL broth (RSB; Difco) at 37°C under 10% CO_2_ for 48 h until they reached an OD540 of 0.6, corresponding to 5 x 10^8^ CFU/ml. The cultures were centrifuged at 10,000 x g for 20 min at 4°C, then the resulting supernatant was removed and sterilised by filtration through a 0.22-µm pore size membrane filter (Millipore; Bedford, MA, USA). Paper disks (8 mm) containing 50 µl of *Lactobacillus* filtrate were placed on the blood agar plates on which the test organisms had been spread. These test plates were incubated at 37°C for 24 h, except for those of *S. mutans* and *S.* *sobrinus*, which were incubated at 37°C under 10% CO_2_ for 48 h, and plates of *P.* *gingivalis*, which were incubated at 37°C under anaerobic conditions for 48 h. Antibacterial activity was determined from the diameter of the inhibitory zone around the paper disk, with the minimum inhibitory zone set at 10 mm. Since *P. gingivalis* was considered a keystone bacterium,^12^ lactic acid bacteria that produced extracellular, soluble antimicrobial agents creating an inhibitory zone of more than 12 mm against *P.* *gingivalis* were analysed further, as described below.

Briefly, the test bacterial strains were grown on the appropriate media for optimal growth and then adjusted to a predetermined optical density, as described above. The rates of growth inhibition of the *Lactobacillus* culture filtrates against test bacteria were determined with liquid cultures in 96-well cell culture plates, using a modification of the method described by De Keersmaecker et al.^10^ Todd Hewitt broth (Becton Dickinson Microbiology Systems; Franklin Lakes, NJ, USA) was used for *S. mutans* and *S. sobrinus* growth, and trypticase soy broth (Becton Dickinson Microbiology Systems) containing hemin and menadione was used for growth of *P. gingivalis*. *Lactobacillus* culture filtrates were concentrated five times using an Amicon Ultra-15 Centrifugal Filter unit (Millipore Sigma; Burlington, MA, USA) and dialysed against 10 mM phosphate buffer for 24 h. Aliquots (10 µl) of each concentrated filtrate were dispensed into 96-well culture plates (Nunc; Naperville, IL, USA) with 90 µl (10^6^ CFU) test bacteria and then cultured for 1–2 days under either aerobic or anaerobic conditions at 37°C. Growth was measured as OD575 using a microtiter plate reader.

### Microbial Characterisation

#### Morphology

The selected isolates were first characterised through Gram and capsule staining.

#### Identification of isolates based on 16S rDNA sequence

Identification of selected isolates to the species level was conducted through 16S rDNA sequence analyses. Bacterial DNA was extracted from each culture isolate using a NucleoSpin Tissue Kit (Macheret-Nagel; Düren, Germany) according to the manufacturer’s instructions. Partial 16S rDNA (approximately 500 bp from the 5’ end) was amplified using universal primers 27F (5’-AGAGTTTGATCCTGGCTCAG-3’) and 520R (5’-ACCGCGGCTGCTGGC-3’). The PCR reaction mixture (20 µl) was composed of 0.1 µl TaKaRa Ex Taq (Takara Bio; Shiga, Japan), 2 µl 10 x Ex Taq Buffer (Takara Bio), 1.6 µl dNTP mixture (2.5 mM each, Takara Bio), 0.2 ml each primer (100 µM), 1 µl template DNA, and 14.9 µl distilled water. The amplification program was 3 min at 95°C; 30 cycles of 95°C for 30 s, 50°C for 30 s, and 72°C for 1 min; and a final extension at 72°C for 10 min. The PCR products were purified using the GFX PCR DNA and Gel Band Purification Kit (Amersham Biosciences; Piscataway, NJ, USA) according to the manufacturer’s instructions. DNA sequence analyses of the purified DNA were performed via the dideoxy chain termination method using the 27F primer, ABI PRIZM BigDye Terminator Cycle Sequencing Kit (Applied Biosystems; Foster City, CA, USA), and an ABI PRIZM 3100 Genetic Analyzer (Applied Biosystems). The sequences were compared to those of reference strains deposited in the DNA Data Bank of Japan using BLAST search. A greater than 99% similarity to the 16S rDNA sequence of a type strain was used as the criterion for identification. The identification of some *Lactobacillus* species with high levels of sequence similarity to closely related species, such as *L. gasseri* and *L. casei*, was further confirmed through 16S-23S rDNA sequence analyses or a multiplex PCR assay with recA gene-derived primers according to the method of Tannock et al.^33^

### Antimicrobial Characterisation

#### pH sensitivity

The selected isolates were grown in RSB adjusted to pH 4, 5, 6, 7, 8, or 9 and incubated at 37°C for 48 h, after which their supernatants were concentrated and dialysed, as described above. Aliquots (10 µl) of each of the concentrated filtrates were examined for antimicrobial activity against *P. gingivalis*. The resulting data are reported as the percentage of growth inhibition compared to the appropriate untreated control group (100%).

#### Effect of enzymes

To determine whether the antimicrobial substance in the culture filtrate obtained from the selected isolate was proteinaceous, the filtrate was treated with pepsin (Sigma Chemicals; St Louis, MO, USA), trypsin (Sigma), and proteinase K (Sigma) for 2 h at 37°C, as described previously.^35^ The final concentrations of the enzymes were 1 mg per ml. To test for the presence of hydrogen peroxide, the filtrates were treated with catalase (final concentration, 0.5 mg per ml) for 1 h at 37°C. After enzymes were added to the culture filtrates and reacted, the enzymes in the filtrates were inactivated by heat treatment at 65°C for 30 min and tested for residual antimicrobial activity. That is, the treated and untreated filtrates, as well as controls containing pepsin, trypsin, and catalase at the appropriate concentrations, were processed as described above.

#### Heat sensitivity

The supernatants of antimicrobial cultures were heated to either 60°C, 80°C, or 100°C for 30 min, or 121°C for 20 min and then processed, as described above.

## Results

### Antimicrobial Screening

**Fig 1:**
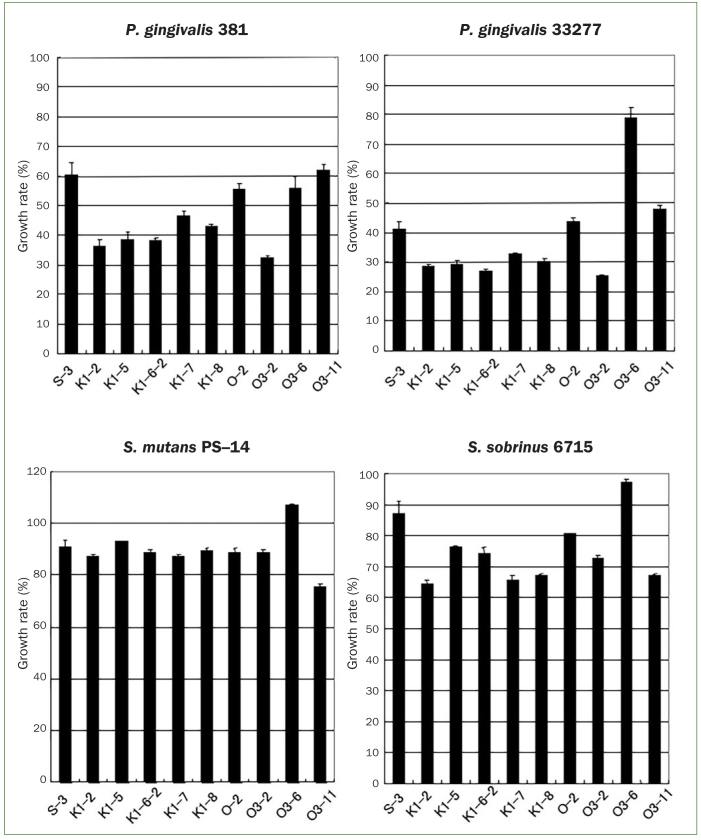
The second antimicrobial screening of the selected antibacterial isolates from the saliva samples (turbidity method). Laboratory stock strains were incubated with the collected culture supernatants prepared from the antibacterial isolates. The results were expressed as the percentage of oral pathogens sensitive to each supernatant specimen. The values shown are means ± SD from the results of 3 experiments.

Of the 200 colonies isolated from the healthy oral cavities of 20 volunteers and then grown in RSB, 122 strains showed primary antibacterial activities based on the paper disk method (Table 1). Among those, 116 strains showed antimicrobial activity against *P. gingivalis* 381 and 122 strains showed activity against *P. gingivalis* 33277. In addition, 94 strains showed antimicrobial activity against *S. sobrinus* 6715 and 48 showed activity against *S. mutans* PS-14. Based on inhibitory zones greater than 12 mm against the periodontopathic bacterium *P. gingivalis*, 10 isolates were selected as having the most significant activity. Using a 96-well plate growth-inhibition screening method, 10 isolates of lactobacilli, designated S-3, K1–2, K1–5, K1–6–2, K1–7, K1–8, O-2, O3–2, O3–6, and O3–11, showed apparent antimicrobial effects against the test strains when grown on RSB. K1–2, K1–5, K1–6–2, K1–7, K1–8, and O3–2 inhibited the growth of *P. gingivalis* 381 by 53.6–67.8%, and of *P. gingivalis* 33277 by 67.1–74.8% (Fig 1).

**Table 1:** First antimicrobial screening of the isolates from saliva (paper disk method)

Test strains	Number of isolates showing the effect towards test strains
*P. gingivalis* 381	116 (58%)
*P. gingivalis* 33277	122 (61%)
*S. mutans* PS-14	48 (24%)
*S. sobrinus* 6715	94 (38%)

Results are expressed as the numbers and percentage of the oral isolates which inhibited each test strain. The antimicrobial test was performed with the disk method.

Disks containing culture filtrates which showed a non-colonised zone around them were interpreted to be antimicrobial.

K1–2, K1–7, K1–8, and O3–11 showed moderate (32.7–35.4%) inhibition of *S. sobrinus* 6715, while O3–11 weakly (24.4%) inhibited *S. mutans* PS-14 (Fig 1). The pH values (pH 4.5–5.1) of the culture filtrates from the isolates were lower than those (pH 4.9–5.7) of culture filtrates from isolates with no or weak antibacterial activities. Therefore, we adjusted the pH of the culture media to the range of 4.0–7.0 and examined the resulting effects on the test strains. However, no significant effects were observed (data not shown). Based on these results, six isolates that demonstrated strong antibacterial activity against *P. gingivalis* (K1–2, K1–5, K1–6–2, K1–7, K1–8, and O3–2) were selected for further characterisation.

### Microbial Characterisation and Identification

K1–2, K1–5, K1–6–2, K1–7, K1–8, and O3–2 were Gram-positive rods without capsules. 16S rDNA sequencing of the bacterial isolates led to identification of K1–2, K1–7, and K1–8 as *Lactobacillus casei*, K1–5 and K1–6–2 as *L. fermentum*, and O3–2 as *L. gasseri*.

### Antimicrobial Characterisation

As shown in Tables 2 to 4, the antimicrobial activities of K1–2, K1–7, K1–8, K1–6–2, K1–5, and O3–2 were affected by catalase, proteolytic enzymes, pH, and temperature. The antimicrobial activities of K1–6–2, K1–5, and O3–2 were reduced (5–14%) by catalase treatment, while that of K1–6–2 was reduced (9%) by trypsin treatment. However, pepsin and proteinase K did not affect the antimicrobial activities of any of the strains (Table 2). Furthermore, although the antimicrobial activities of the tested isolates were hardly affected by heat treatment at 60°C, their activities were reduced by 10–31% after treatment at 100°C and by 22–40% following autoclave treatment (Table 3). The activities of KI-8 and K1–6–2 were reduced after treatment at pH values below 5, while those of K1–2, K1–7, and O3–2 were reduced after treatments with pH values below 5 and above 7. Notably, the antimicrobial activity of K1–2 was completely inactivated after treatment at pH 9.

**Table 2:** Effect of enzymes on the antibacterial activity of selected oral isolates

Oral isolates	Control	Catalase	Trypsin	Pepsin	Proteinase K
K1-2	100.0	102.0 ± 2.1b	104.2 ± 1.2b	100.0 ± 1.4b	105.1 ± 2.0b
		104.4 ± 1.6c	110.2 ± 1.8c	114.1 ± 2.0c	116.5 ± 1.5c
K1-7	100.0	102.2 ± 2.8b	109.5 ± 2.0b	100.0 ± 0.7b	111.5 ± 2.4b
		104.8 ± 2.1c	110.1 ± 4.2c	108.1 ± 2.9c	126.4 ± 3.1c
K1-8	100.0	98.1 ± 1.8b	105.1 ± 3.8b	100.0 ± 1.5b	122.2 ± 2.2b
		102.4 ± 2.0c	102.6 ± 2.4c	107.7 ± 4.3c	118.4 ± 3.6c
K1-6-2	100.0	93.2 ± 1.7b	100.0 ± 1.9b	100.0 ± 2.0b	106.3 ± 1.8b
		95.0 ± 1.2c	94.2 ± 1.3c	104.3 ± 3.2c	120.9 ± 4.5c
K1-5	100.0	93.2 ± 1.7b	100.0 ± 1.9b	100.0 ± 2.0b	106.3 ± 1.8b
		95.0 ± 1.2c	94.2 ± 1.3c	104.3 ± 3.2c	120.9 ± 4.5c
O3-2	100.0	86.7 ± 2.9b	107.8 ± 5.1b	100.0 ± 1.8b	100.0 ± 1.8b
		95.3 ± 2.7c	100.0 ± 3.1c	104.9 ± 2.9c	106.0 ± 1.4c

Results are expressed as the percentage of the residual activity compared to the control (PBS). Data are expressed as the mean ± SEM of three different experiments. *P. gingivalis* 381 (b) and *P. gingivalis* 33277 (c) were used as the test strains.

**Table 3:** Effect of heat on the antibacterial activity of selected oral isolates

Oral isolates	Control	60°C, 30 min	100°C, 30 min	121°C, 30 min
K1-2	100.0	100.0 ± 2.0b	81.0 ± 1.7b	60.3 ± 2.2b
		93.8 ± 3.0c	87.7 ± 2.9c	64.6 ± 2.6c
K1-7	100.0	97.1 ± 1.5b	75.3 ± 2.2b	63.1 ± 1.4b
		101.9 ± 2.4c	80.7 ± 1.8c	73.6 ± 2.4c
K1-8	100.0	94.1 ± 1.2b	74.5 ± 1.8b	62.4 ± 1.7b
		94.8 ± 1.6c	69.6 ± 1.1c	63.8 ± 1.0c
K1-6-2	100.0	99.2 ± 1.8b	90.4 ± 1.9b	78.2 ± 2.1b
		94.5 ± 1.5c	84.9 ± 1.5c	69.8 ± 1.8c
K1-5	100.0	98.2 ± 2.0b	90.6 ± 2.0b	75.4 ± 1.5b
		94.4 ± 1.8c	79.1 ± 2.8c	63.9 ± 1.4c
O3-2	100.0	86.7 ± 2.9b	107.8 ± 5.1b	100.0 ± 1.8b
		95.3 ± 2.7c	100.0 ± 3.1c	104.9 ± 2.9c

Results are expressed as the percentage of the residual activity compared to the control (non-heat). Data are expressed as the mean ± SEM of three different expreriments. *P. gingivalis* 381 (b) and *P. gingivalis* 33277 (c) were used as the test strains.

**Table 4:** Effect of pH on the antibacterial activity of selected oral isolates

Oral isolates	Control	pH4	pH5	pH6	pH7	pH8	pH9
K1-2	100.0	80.6 ± 2.1b	93.5 ± 1.3b	102.9 ± 2.5b	88.0 ± 1.6b	73.1 ± 0.8b	0 ± 0b
		81.5 ± 1.6c	96.1 ± 2.0c	96.9 ± 1.9c	81.3 ± 1.7c	71.2 ± 1.1c	36.9 ± 0.6c
K1-7	100.0	84.6 ± 0.9b	99.2 ± 2.4b	112.3 ± 2.9b	136.9 ± 4.4b	69.2 ± 0.8b	78.4 ± 1.2b
		74.6 ± 1.1c	93.6 ± 1.9c	98.5 ± 2.0c	71.8 ± 3.9c	54.7 ± 0.5c	51.2 ± 0.9c
K1-8	100.0	85.3 ± 0.8b	98.8 ± 1.8b	149.2 ± 4.2b	141.2 ± 4.1b	126.9 ± 2.8b	141.2 ± 3.6b
		83.0 ± 1.6c	94.7 ± 1.5c	152.5 ± 3.4c	115.2 ± 3.7c	105.0 ± 2.0c	122.0 ± 3.0c
K1-6-2	100.0	83.1 ± 2.1b	96.2 ± 2.0b	138.5 ± 3.3b	120.0 ± 1.8b	116.9 ± 2.1b	124.6 ± 2.9b
		84.7 ± 1.5c	89.8 ± 1.9c	142.3 ± 3.8c	115.1 ± 2.5c	91.5 ± 1.7c	110.1 ± 2.1c
K1-5	100.0	96.8 ± 2.8b	100.2 ± 2.2b	141.9 ± 4.0b	129.0 ± 4.2b	125.8 ± 2.4b	122.6 ± 2.0b
		100.0 ± 1.8c	91.5 ± 2.8c	144.1 ± 3.2c	112.2 ± 3.0c	91.5 ± 1.5c	111.8 ± 1.6c
O3-2	100.0	79.7 ± 4.0b	94.0 ± 1.2b	117.4 ± 1.7b	115.9 ± 2.7b	100.2 ± 0.8b	100.0 ± 1.7b
		67.5 ± 3.2c	87.5 ± 1.8c	93.8 ± 1.1c	68.7 ± 1.2c	51.3 ± 1.2c	45.0 ± 0.8c

Results are expressed as the percentage of the residual activity compared to the control (non-treated). Data are expressed as the mean ± SEM of three different experiments. *P. gingivalis* 381 (b) and *P. gingivalis* 33277 (c) were used as the test strains.

## Discussion

Generally, *Lactobacillus* species are believed to play positive roles in maintaining good health and immune system function in humans. We isolated 200 putative *Lactobacillus* strains from the oral cavities of 20 healthy volunteers. Among them, 116-122 strains showed antibacterial effects against *P. gingivalis* and 48–94 strains showed antibacterial effects against *S. mutans* and *S. sobrinus*. Six isolates produced antimicrobial substances that inhibited a keystone pathogen^12^ such as *P. gingivalis*; however, these strains did not show notable antibacterial effects against *S. mutans* or* S. sobrinus*. A previous study showed that *Lactobacillus* strains from periodontally healthy patients exhibited less antimicrobial activity against *S. mutans* than strains from patients with chronic periodontal disease.^18^ Our results support these findings and may also explain the inverse association between caries and periodontal disease observed by Sioson et al,^30^ at least partly. This study demonstrated that oral lactobacilli also possess antimicrobial activities, as previously reported for intestinal and vaginal lactobacilli.^11,25,26^ Oral *Lactobacillus* species were found to constitute a portion of the intestinal lactobacilli community.^9^ Therefore, antimicrobial substances produced by oral lactobacilli may also be effective against pathogenic bacteria in the intestine.

*Lactobacillus* strains produce organic acids such as lactic acid, which can contribute to antimicrobial activities against certain microorganisms.^32^ Therefore, we assumed that the antimicrobial substances produced by isolates of oral bacteria in this study may have included organic acids, although organic acid production in the oral cavity increases the risk for dental caries. We also showed that the antimicrobial activities of the isolates were greater at neutral pH than at acidic and alkaline pH levels. Furthermore, when the pH of the medium was adjusted to match the pH of culture filtrates, there was no effect on the antimicrobial activities of laboratory test strains. These findings indicate that the antimicrobial substances in the present oral isolates were not organic acids. Therefore, these strains may be useful as potential oral probiotics. *Lactobacillus* species produce hydrogen peroxide. The antimicrobial activities of some oral isolates were slightly reduced after treatment with catalase, which suggests that a few of the isolates were able to produce hydrogen peroxide, another antimicrobial substance.^24^ However, because the suppression of antibacterial activity by catalase treatment was minor (7–14%), it is reasonable to conclude that hydrogen peroxide was not the main active antimicrobial factor in the filtrates. *Lactobacillus* can also produce bacteriocins.^8,15,36^ For example, salivacin 140 from *L. salivarius*,^2^ plantaricin 423 from *L. plantarum*,^36^ and acidocin JI229 from *L. acidophilus*^31^ are bacteriocins that are reportedly resistant to heat. The antimicrobial activity of lactic acid bacteria isolated also maintained antibacterial activity of 94% or more by heat treatment at 60°C and that of 75% or more even at heat treatment at 100°C, although this phenomenon may be clinically meaningless for use as probiotics. Here, the antibacterial activities of the isolated strains were resistant to treatments with trypsin, pepsin, and proteinase K, with one exception. Indeed, several authors have shown that bacteriocins can be resistant to degradation by proteases.^3,14,28^ Thus, the antibacterial activities of strains we isolated may be due to protease-resistant bacteriocins. Although bacteriocins are antimicrobial agents with a relatively narrow spectrum, some intestinal lactobacilli produce non-bacteriocin antimicrobial substances that are active against both Gram-negative and Gram-positive bacteria in vitro. For example, cell-free supernatants sampled from *L. acidophilus* LA1 and *L. acidophilus* LB were found to contain non-bacteriocin and non-lactic acid antibacterial molecule(s) that were heat stable and insensitive to pronase.^4,7^ Some non-bacteriocin and non-lactic acid antibacterial substances produced by *Lactobacillus* are already known, such as diacetyl and reuterin.^20^ Because the types of antimicrobial substances produced by *Lactobacillus* isolated from oral cavity samples are of great interest, further research is needed.

Probiotic strains are expected to function better in environments similar to the environment from which they were isolated. Therefore, strains isolated from the oral cavity of healthy volunteers are expected to survive with oral pathogens and to be capable of adhering to the oral mucosa.^6^

In the present study, 16S rDNA sequence analyses were used to identify three isolates as *L. casei*, two isolates as *L. fermentum*, and one isolate as *L. gasseri*. Because these species are common components of the normal flora in the human mouth and intestine, and were isolated from the oral cavities of the healthy volunteers in our study, these isolates are promising probiotics that may contribute to the health of the host by improving the equilibrium of flora in the oral cavity and intestine.

## Conclusion

Lactic acid bacteria isolated from the oral cavities of 20 healthy volunteers exhibited antimicrobial activity against *P. gingivalis* with different properties from existing bacteriocins. These bacteria are potential antagonistic probiotics against the periodontal pathogen *P. gingivalis*.
